# Chyluria in a Postpartum Obese Female Patient

**DOI:** 10.7759/cureus.38940

**Published:** 2023-05-12

**Authors:** Vengatesan Kowshik, Swathy Moorthy, Lakshmi Marappa, Basith Ahmed, Emmanuel Bhaskar

**Affiliations:** 1 Internal Medicine, Sri Ramachandra Institute of Higher Education and Research, Chennai, IND

**Keywords:** obesity-related illnesses (oris), lymphatic obstruction, technetium scintigraphy, post partum, chyluria

## Abstract

Chyluria characterized by the passage of milky white urine is rarely encountered these days due to the overall reduction in the number of cases of lymphatic filariasis. Though lymphatic filariasis accounts for the majority of cases of chyluria, nonparasitic causes have also been reported. Case reports of chyluria as a complication in pregnancy have been published but chyluria presenting solely as a postpartum complication has rarely been documented.

We present a case of a 29-year-old female with no known prior comorbidities, who presented with recurring complaints of the painless passage of milky white urine over the last year. Symptoms seem to have started six months post-delivery of her second child. The patient claimed significant weight gain during an otherwise normal pregnancy. She was well-built and had a BMI of 32 kg/m^2^. Her systemic examination and baseline laboratory workup were within normal limits. Postprandial urine was milky white, rich in chylomicrons, with urine chylomicrons of 112 mg/dl. The patient was screened for filariasis, which was negative. An ultrasound of the abdomen was done to rule out the presence of a fistula, but no evidence of one was found on imaging. Tc-99m sulfur colloid scintigraphy revealed an area of abnormal tracer accumulation in the abdomen with the passage of the tracer in the urine container, confirming the presence of chyluria. The patient was recommended to undergo conservative management with dietary modification and weight reduction. She has been closely followed up and has achieved spontaneous resolution of the chyluria. Most patients with chyluria show a good response to conservative management alone as in our case. Surgical intervention is usually indicated for cases not responding to conservative management or for refractory chyluria.

## Introduction

Chyluria as evidenced by the passage of milky white urine represents an abnormal fistulous connection between the lymphatic system and the urinary tract. The milky white nature of urine is due to the presence of chyle, a lymphatic fluid rich in chylomicrons. Chyle normally drains via intestinal lacteals into the thoracic duct and left subclavian vein. An abnormal fistulous connection between this drainage pathway and the urinary tract can result in chyluria. Though often secondary to parasitic infections (lymphatic filariasis caused by Wuchereria bancrofti, Brugia malayi, Brugia timori, etc.), nonparasitic causes have also been found albeit less commonly. Nonparasitic causes include congenital lymphatic malformations, post-surgical lymphourinary fistulas, and obstruction to lymph flow with resultant fistula formation by either malignancy, abscesses, pregnancy, etc. [1,2]. Pregnancy is a rare cause of nonparasitic chyluria where the enlarging uterus obstructs the lymphatic flow resulting in the formation of a lymphourinary fistula [3]. Even in pregnancy, filarial etiology is usually the most common cause of chyluria. Whether of parasitic or nonparasitic etiology, the initial approach to treatment is usually conservative with weight reduction, dietary modification, and/or sclerotherapy. Mahmood et al. in their study observed a significant resolution in chyluria with conservative management alone [4]. Similar results were observed by Tan et al. in their study [5]. Refractory cases alone require surgical interventions like lymphatic dissection or the creation of lymphangiovenous anastomosis. Anthelmintic drugs like diethylcarbamazine, though beneficial in parasitic chyluria, are generally avoided in pregnancy due to the risk of teratogenic side effects. Chyluria presenting solely as a late complication of pregnancy has rarely been documented and we discuss such a case in this report.

## Case presentation

A 29-year-old female with no known prior comorbidities presented to our outpatient department with complaints of the intermittent passage of milky white urine over the last year. The passage of milky white urine had been insidious in onset, initially occasional with normal voids, and had gradually progressed to a state where she was voiding only milky white urine with occasional normal voids. There was no associated history of hematuria, abdominal pain, fever with chills, trauma, swelling of legs, rash, facial puffiness, weight loss, or recent surgery. The patient had been treated with short courses of antibiotics during the initial days of illness, suspecting a urinary tract infection. She consumed a mixed diet. Her obstetric history was P2L2 (para 2, live births 2) with two normal spontaneous vaginal deliveries. During the last pregnancy, the patient had gained a significant amount of weight. Six months postpartum, the patient had started developing the above-mentioned complaints. There was no history of overbearing or prolonged labor during the last pregnancy. There were no similar complaints among the family members. On examination, the patient was well-built with a BMI of 32 kg/m^2^. Her clinical examination was unremarkable except for obesity and the presence of Acanthosis nigricans on general examination. Her baseline investigations are presented in Table 1.

**Table 1:** Baseline blood investigations

Lab parameter	Patient value	Reference range
Hemoglobin	12.8 gm/dl	12–15 gm/dl
Total leucocyte count	7900 cubic mm	4000–11000 cubic mm
Polymorph percentage	67.6%	45–70%
Lymphocyte percentage	19.4%	25–40%
Eosinophil percentage	2.8%	1–6%
Blood urea nitrogen	6 mg/dl	7.9–20.1 mg/dl
Creatinine	0.6 mg/dl	0.7–1.1 mg/dl
Total protein	6.6 gm/dl	6.4–8.2 gm/dl
Serum albumin	4 gm/dl	3.2–4.8 gm/dl
Serum globulin	2.6 gm/dl	2–3.5 gm/dl
Free thyroxine	1.04 ng/dl	0.8–1.8 ng/dl
Thyroid-stimulating hormone	2.20 µIU/ml	0.35–4 µIU/ml
Total cholesterol	149 mg/dl	0–200 mg/dl
Triglycerides	166 mg/dl	0–150 mg/dl
High-density lipoprotein	38 mg/dl	85–60 mg/dl
Low-density Lipoprotein	98 mg/dl	0–100 mg/dl

**Figure 1:**
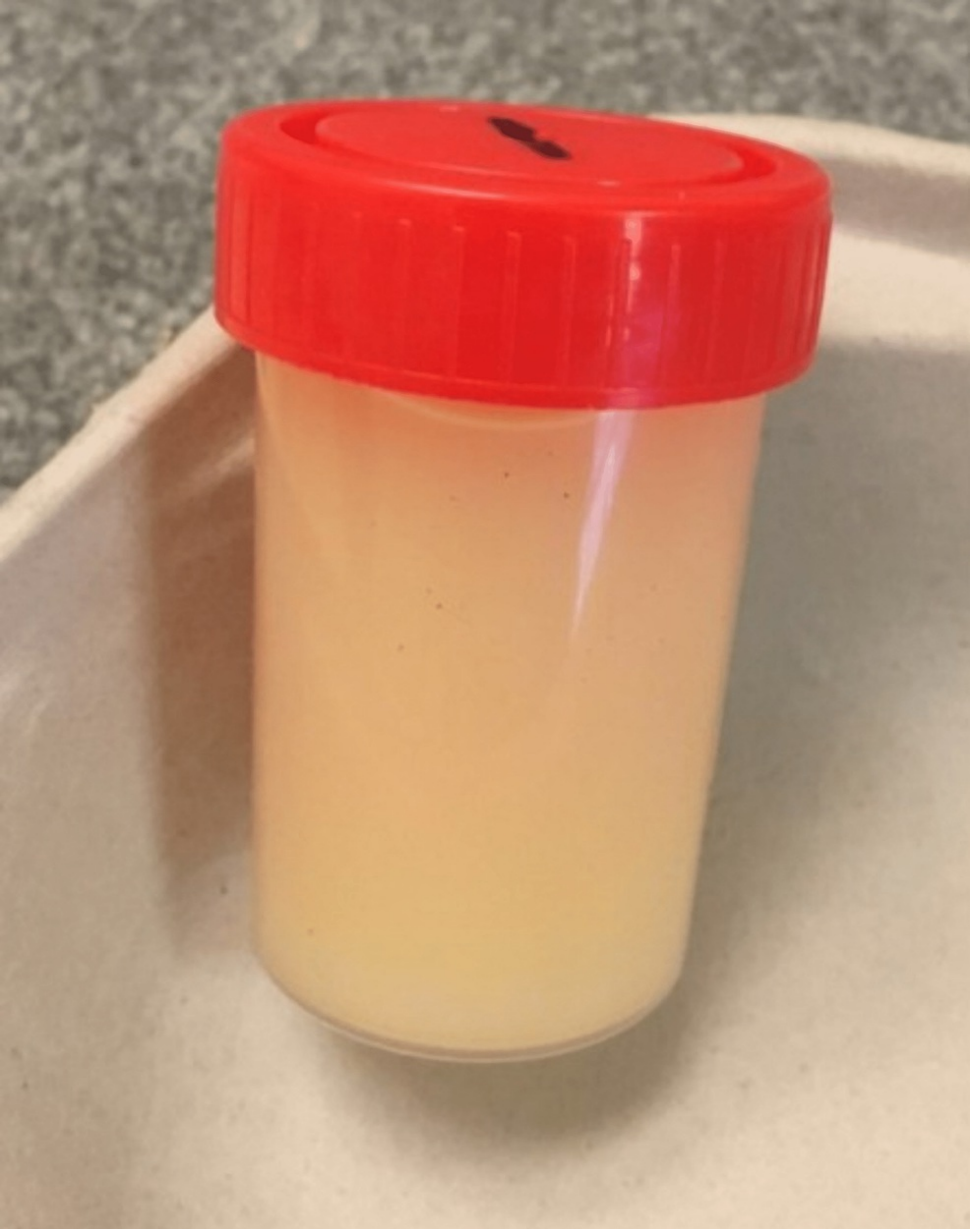
Milky white postprandial urine collected by the patient

The urine sample collected was milky white in color (Figure 1).

**Figure 2:**
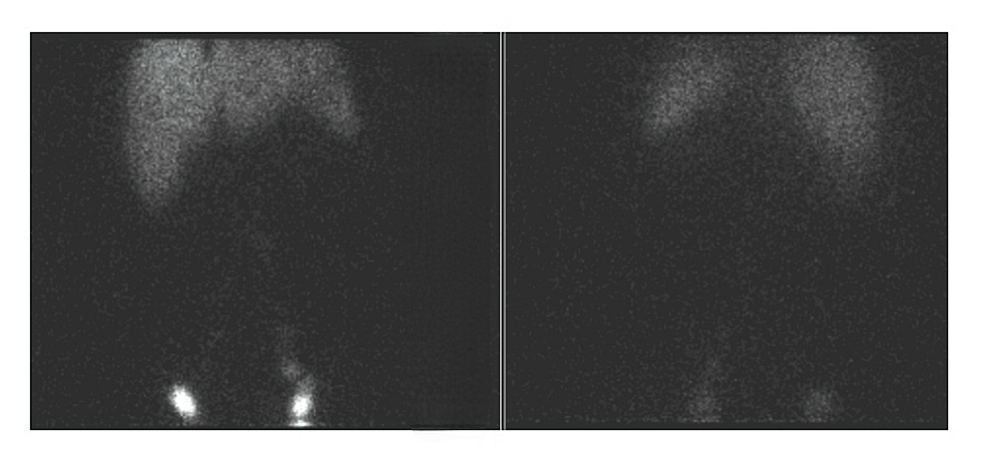
Tc-99m sulfur colloid scintigraphy The image shows the flow of radiotracer in the lymphatic channels after intradermal injection

**Figure 3:**
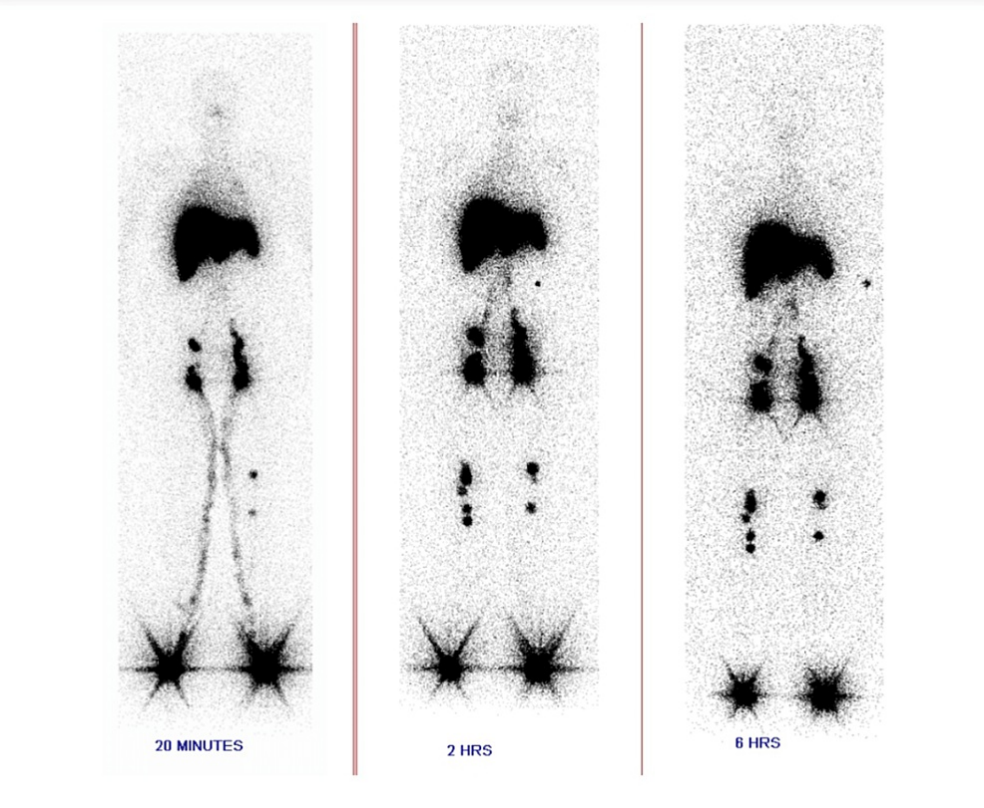
Normal flow of tracer through lymphatic channels after 20 minutes, 2 hours, and 6 hours

**Figure 4:**
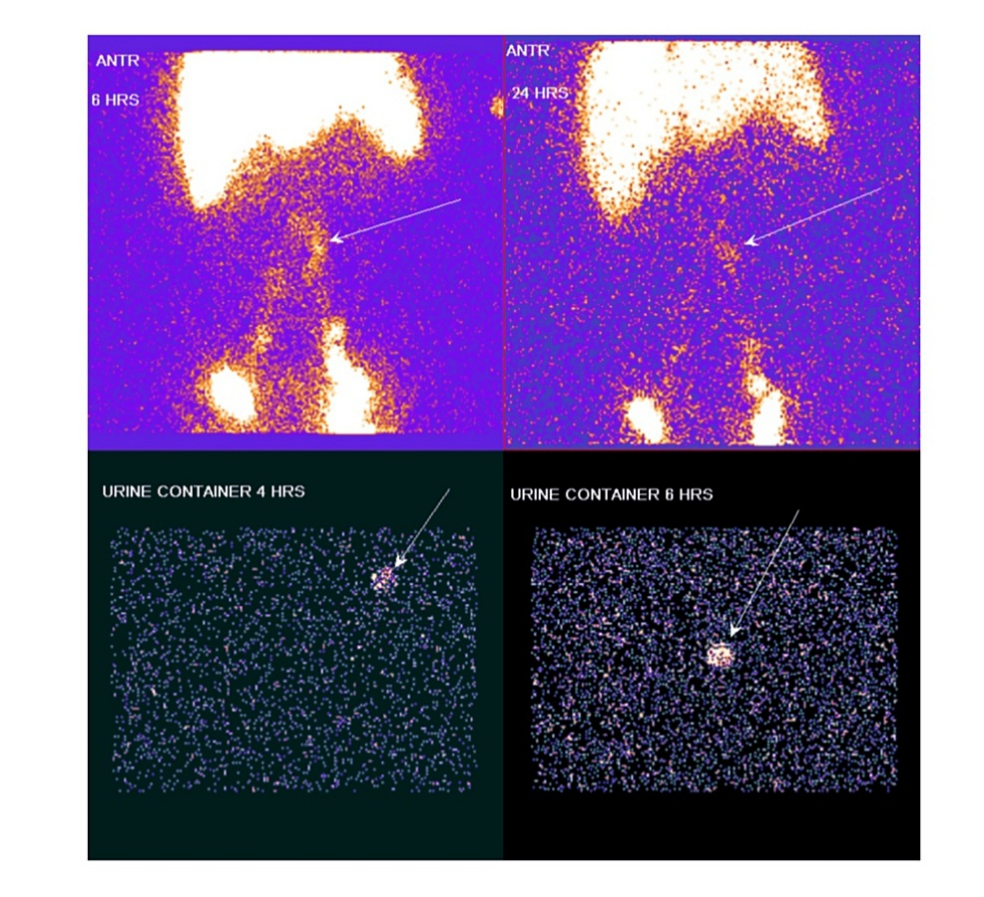
Area of abnormal tracer accumulation in the abdomen (arrows in the upper half of the image) suggestive of obstruction in lymphatic flow. Tracer excretion in urine (arrows in the lower half of the image) confirming the presence of chyluria

Urinalysis revealed acidic urine with pH 5.0, containing 2+­ proteinuria and corresponding to protein loss <100 mg/dl, 2-4 pus cells/high power field with no casts or crystals. Protein quantification was done, which revealed a urine protein-creatinine ratio of 1.40 (normal value: <0.2), urine spot protein of 45.2 mg/dl (normal value: <150 mg/dl), and urine spot creatinine of 33 mg/dl (normal range: 37-250 mg/dl). Urine culture revealed no growth. Urine was abundant with chylomicrons - urine spot chylomicrons measured 112 mg/dl, favoring the presence of chyluria. As part of the chyluria workup, the patient was screened for lymphatic filariasis (identification of microfilariae on blood smears), which was negative. Ultrasound of the abdomen was done to look for evidence of a lymphatic-urinary fistula. It revealed only grade 1 fatty liver with a mildly dilated left pelvicalyceal system, likely due to an overdistended bladder. With ultrasound imaging being inconclusive, the patient was then evaluated with lymphoscintigraphy. Tc-99m sulfur colloid scintigraphy was done. Figures 2-3 show the normal flow of the tracer in the lymphatic channels. Figure 4 reveals an area of abnormal tracer accumulation in the abdomen (arrows in the upper half of the image suggesting obstruction to lymphatic flow) with tracer concentration in the urine container (arrows in the lower half of the image), confirming the presence of chyluria.

The patient was put on a modified diet (restriction of fatty foods, substitution with medium-chain fatty acids) and advised weight reduction and daily exercises. The patient responded well to conservative management. Her BMI dropped to 24 kg/m^2^ and she had no further chyluric episodes on serial follow-ups.

## Discussion

While chyluria characterized by the intermittent discharge of intestinal lymph (chyle) into the renal pelvis and urine is usually considered a harmless condition, if left untreated, it can lead to fatal outcomes. Various theories have been proposed regarding the development of chyluria; currently, two theories have been mainly highlighted as an explanation of the causative factors (obstructive and regurgitative theories) [6-7]. Chyluria, unless otherwise proven, is usually of parasitic origin; 95% of parasitic causes are attributed to Wuchereria bancrofti, Brugia malayi, and Brugia timori with the remaining 5% secondary to Taenia echinococcus, Taenia nana, Ankylostomiasis, and Trichiniasis [8]. Blockage of the major retroperitoneal lymphatics and thoracic duct by the mature parasite heralds the subsequent development of urinary fistulae and the occurrence of chyluria. When associated with a parasitic cause, cases can also present with concomitant genital manifestations, cellulitis, abscesses, and hematuria. Nonparasitic causes are rarer and are almost always nontropical.

Chyluria in pregnancy has been well discussed but chyluria presenting solely as a postpartum complication, as in our case, has not been documented previously. Patients classically manifest a worrying appearance of milky white urine, which can be associated with dysuria, urgency, or urinary retention secondary to chylous clots. Long-standing chyluria in both pregnant and nonpregnant populations can be associated with hypoproteinemia, edema, cachexia, weight loss, and opportunistic infections secondary to deficiencies of IgA and IgG immunoglobulins. Similar to the Date study [9], Ciferri et al. also found significant immunological disturbances in patients with chronic chyluria [10], which could be explained by the loss of lymphatic humoral and cellular elements. The diagnosis of chyluria can be confirmed by evaluating a sample of postprandial urine for chylomicrons and triglycerides [11,12]. Other differentials to be considered when encountering white urine include phosphaturia, amorphous urates, severe pyuria, lipiduria, and caseousuria due to renal TB. Chylous urine is usually acidic, which, on sedimentation, forms three layers, is rich in triglycerides, turns transparent on adding ether, and becomes bright orange with the addition of Sudan III stain. Tools that might aid the evaluation of parasitic causes include the presence of eosinophilia, immunochromatographic tests for filaria, urine for acid-fast bacilli, or evidence of urinary tract infection. Once parasitic causes have been ruled out, nonparasitic causes have to be investigated; ultrasound abdomen and pelvis as a first-line imaging tool might help look for evidence of lymphatic-urinary fistula. If ultrasound imaging is inconclusive, fistula can be investigated by higher radiological modalities like CT or MRI or by cystoscopy, retrograde pyelography, or lymphoscintigraphy, which has therapeutic benefits as well as acts as a sclerosing agent.

There is currently no universally accepted grading system for chyluria. Available scales grade chyluria as mild, moderate, and severe based on the degree of chyluria, associated symptomatology, episode frequency, and the extent of calyceal involvement [13]. Most cases of chyluria can be managed initially with dietary modifications and weight reduction alone. The proposed hypothesis is that weight control reduces the compressive effect over the lymphatics and dietary modification in the form of substitution of long-chain fatty acids with medium-chain fatty acids and helps bypass the lacteals due to the direct absorption of medium-chain fatty acids into the portal vein. Abdominal binders may be used, which increases the abdominal pressure and reduces lympho-urinary reflux. Case reports suggesting the beneficial role of somatostatin analogs and ACE inhibitors in the management of chyluria have also been published [14,15]. In the case of parasitic chyluria, patients usually respond well to diethylcarbamazine. In cases of failed medical therapy, refractory severe chyluria with recurrent colics, and ill health due to immune suppression, surgical intervention is recommended. Available surgical interventions include (a) endoscopic sclerotherapy (EST) with either silver nitrate, povidone-iodine, or bromide; (b) surgical lymphatic dissection; and (c) microsurgery. Patients require six-monthly urine evaluations for chyle. Our patient showed spontaneous resolution of symptoms with dietary modification and weight reduction alone. Our findings correlated with those of the study by Singh et al., in which the relationship between quantity and type of dietary fat and the degree of loss of lipids in urine was studied [16].

## Conclusions

Nonparasitic causes of chyluria, though uncommon, must always be evaluated in patients presenting with chyluria. In most cases, conservative management with weight reduction and dietary modification alone is enough for the complete resolution of symptoms. Surgical intervention is usually reserved for refractory cases or for patients in ill health.
